# Psychosocial risk profiles to address future health emergencies: a country study during the COVID-19 lockdown period in Colombia

**DOI:** 10.3389/fpubh.2024.1323490

**Published:** 2024-03-22

**Authors:** Martha Milena Bautista-Gomez, Anthony Constant Millán De Lange, Jorge Enrique Palacio Sañudo, Laura Sofia Zuluaga, Daniel Alfonso Bolívar Pimiento, Yesith Guillermo Toloza Perez, Jeadran Malagon-Rojas, Marcela Maria Mercado-Reyes, Martha Ospina

**Affiliations:** ^1^Centro Internacional de Entrenamiento e Investigaciones Médicas (CIDEIM), Cali, Colombia; ^2^Universidad Icesi, Cali, Colombia; ^3^Universidad del Norte, Barranquilla, Colombia; ^4^Department of Psychology, Instituto Nacional de Salud de Colombia (INS), Bogotá, Colombia; ^5^Faculty of Natural Sciences and Engineering, Universidad Jorge Tadeo Lozano, Bogotá, Colombia

**Keywords:** psychosocial impact, COVID-19, health emergency, mental health, social vulnerability

## Abstract

**Introduction:**

The different strategies used worldwide to curb the COVID-19 pandemic between 2020 and 2021 had a negative psychosocial impact, which was disproportionately higher for socially and economically vulnerable groups. This article seeks to identify the psychosocial impact of the confinement period during the COVID-19 pandemic for the Colombian population by identifying profiles that predict the levels of different mental health indicators (feelings of fear, positive emotions or feelings during free time, and work impact) and based on them, characterize the risk factors and protection that allows us to propose guidelines for prevention or recovery from future health emergencies.

**Methods:**

This is an observational, cross-sectional, retrospective *ex post facto* study. Multistage cluster probabilistic sampling and binary logistic regression analysis were used to predict extreme levels of various mental health indicators based on psychosocial indicators of the COVID-19 confinement period and to identify risk and protection factors,

**Results:**

A relationship was established between the combination of some of the different psychosocial factors evaluated (this combination being the predictive profile identified) with each of the three main variables: feeling of fear (*n* = 8,247; *R* = 0.32; *p* = 0.00; P_overall_ = 62.4%; 𝜔_overall_ = 0.25; 1-𝛽_overall_ = 1.00), positive emotions or feelings during free time (*n* = 6,853; *R* = 0.25; *p* = 0.00; P_overall_ = 59.1%; 𝜔_overall_ = 0.18; 1-𝛽_overall_ = 1.00) and labour impact (*n* = 4,573; *R* = 0.47; *p* = 0.63; P_overall_ = 70.4%; 𝜔_overall_ = 0.41; 1-𝛽_overall_ = 1.00), with social vulnerability determined by sociodemographic factors that were common in all profiles (sex, age, ethnicity and socioeconomic level) and conditions associated with job insecurity (unemployed, loss of health insurance and significant changes to job’s requirements) and place of residence (city).

**Conclusion:**

For future health emergencies, it is necessary to (i) mitigate the socio-employment impact from emergency containment measures in a scaled and differentiated manner at the local level, (ii) propose prevention and recovery actions through psychosocial and mental health care accessible to the entire population, especially vulnerable groups, (iii) Design and implement work, educational and recreational adaptation programs that can be integrated into confinement processes.

## Introduction

According to the theoretical model of social psychology (1), which accentuates the significance of social factors in the onset of mental issues, when an individual encounter an exceedingly potent external stressor, such as a pandemic, their mental well-being may deteriorate. In this regard, the alterations brought about by the COVID-19 pandemic in people’s lives have given rise to fresh psychosocial impact risks for their health and overall welfare. These include the apprehension of contagion, social isolation, heightened demands for digitalization, job instability, an increased susceptibility to violence, and an imbalance between work and personal life, among others (2).

The various approaches employed globally to mitigate the COVID-19 pandemic between 2020 and 2021 had an adverse social effect as these measures affected employment and people’s means of subsistence. On a global scale, the International Labour Organization (ILO) disclosed that, during the initial year of the pandemic, 13 million individuals found themselves unemployed (3). Similarly, within Latin America, poverty rates escalated, exacerbating the overall vulnerability of families (4), and notable detriments to the mental well-being of the populace became apparent (5, 6). Nonetheless, these detrimental consequences were notably more pronounced among socially and economically disadvantaged segments (7).

In Colombia, a national study (18,472 people from 10 cities) of SARS-CoV-2 seroprevalence was conducted between September and December 2020 (8). In a first analysis of the results, seropositivity varied strongly among cities, which was explained mostly by socioeconomic factors, followed by ethnicity, education level, and family composition (9). A second analysis focusing on children and young people revealed that this group was particularly vulnerable, as were people who lived in cities with low social development indicators, because of a higher risk of infection by SARS-CoV-2 (10).

Building upon the findings of prior investigations, this article presents an in-depth analysis of the psychosocial ramifications stemming from SARS-CoV-2, utilizing data sourced from a national study conducted in Colombia (8). In this study, we examined the primary stressors prevalent during the period of lockdown, which can be summarised as follows: (i) The profound apprehension of losing one’s own life or that of a family member, signifying the fear, anxiety, stress, and depression experienced by the population due to COVID-19 (11). (ii) The variable of job insecurity, defined as a condition where individuals lack the certainty that their employment, their primary source of income, will remain stable (11). (iii) The stress or anxiety associated with either an inadequate use of newfound free time, an uncommon occurrence in daily life, or the inclusion of novel activities and leisure pursuits, such as sports and physical activities, digital media consumption, artistic endeavours, and socially engaging pastimes, among others (12). These three variables were scrutinized in connection with socioeconomic vulnerability, which is comprehended as the insecurity, vulnerability, and exposure faced by communities, families, and individuals in their living conditions as a consequence of an event or threat (13, 14). In such circumstances, adverse socioeconomic factors constrain their capacity to anticipate, combat, withstand, and recover from the impact of that event or threat (15, 16).

In this vein, the aim of this article is to contribute to a better understanding of the impact of the confinement period during the SARS-CoV-2 pandemic on psychosocial impact of the Colombian population. With this approach, the objective is to generate risk profiles that report the variables that predict such outcomes to determine the profiles that present the highest risk factors and, also this research seeks to propose guidelines based on these profiles for the prevention or recovery from future health emergencies.

## Methods

### Type of study

This was an observational (17), cross-sectional, *ex post facto* retrospective study (18).

### Population

The study population was noninstitutional civilians older than 18 years residing in the municipal seats of Bogotá, Barranquilla, Bucaramanga, Cali, Cúcuta, Medellín, Villavicencio, Leticia, Ipiales, and Guapi. who were included in the Colombian National study of SARS-CoV-2 seroprevalence (8).

### Sample and sampling

The sampling frame was established from the list of dwellings, households, people, and cartographic inventory of the selected cities from the census of Departamento Administrativo Nacional de Estadística (National Administrative Department of Statistics, DANE). Multistage cluster sampling was employed. The sample sizes were calculated for a prevalence of 30% with marginal sampling errors of 3.0% for Bogota, Cali, and Medellin, 3.5% for Leticia, Barranquilla, Bucaramanga, Cúcuta, Villavicencio, and Ipiales, and 5.0% for Guapi and regions with geographically restricted access, which are equivalent to relative errors of 5, 6, and 8.5%, respectively. Details about the sampling method can be found in Mercado et al. (9). Descriptive characteristics of the sample are provided in Table 1.

**Table 1:** Descriptive data.

Variable		Complete sample
General		
Sex*	Male	38.5% (*n* = 6,928)
	Female	61.5% (*n* = 11,067)
According to your culture, town, or physical traits, you identify as:	Mestizo	52.1% (*n* = 8,910)
	White	20.2% (*n* = 3,454)
	Black	7.43% (*n* = 1,270)
	Indigenous	4.58% (*n* = 784)
	Other	7.43% (*n* = 1,271)
	Do not know	8.26% (*n* = 1,413)
What is your current marital status?	Single	51.22% (*n* = 9,097)
	Married	20.95% (*n* = 3,722)
	Common-Law Marriage (Free union)	20.54% (*n* = 3,649)
	Widowed	3.88% (*n* = 689)
	Divorced	3.41% (*n* = 605)
Over the past 12 months, what was your main occupation?	Unemployed	8.26% (*n* = 1,449)
	Employed	22.61% (*n* = 3,965)
	Independent	18.7% (*n* = 3,279)
	Student	22.66% (*n* = 3,974)
	Informal worker	2% (*n* = 350)
	Housewife	20.88% (*n* = 3,661)
	Pensioner	4.64% (*n* = 813)
	Health personnel	0.26% (*n* = 45)
What is the highest education level you have achieved? Main occupation?	None	1.94% (*n* = 344)
	Preschool	4.09% (*n* = 724)
	Primary	27.37% (*n* = 4,846)
	Secondary	36% (*n* = 6,373)
	Technical or technological	15.33% (*n* = 2,714)
	Undergraduate	11.48% (*n* = 2032)
	Post-graduate	3.8% (*n* = 672)
What is your health insurance regime?**	Contributory	52.16% (*n* = 9,187)
	Subsidized	39.38% (*n* = 6,936)
	Special or exception	2.71% (*n* = 477)
	Not insured	5.5% (*n* = 968)
	Indeterminate/pending	0.26% (*n* = 46)
City	Barranquilla	8.15% (*n* = 1,674)
	Bogotá	24.02% (n = 4,933)
	Bucaramanga	8.84% (*n* = 1815)
	Cali	10.72% (*n* = 2,201)
	Cúcuta	8.64% (*n* = 1774)
	Guapi	4.35% (*n* = 894)
	Ipiales	8.06% (*n* = 1,656)
	Leticia	8.26% (*n* = 1,697)
	Medellín	11.21% (*n* = 2,302)
	Villavicencio	7.74% (*n* = 1,589)
Home		
Type of dwelling	House	23.68% (*n* = 4,775)
	Apartment	36.98% (*n* = 7,456)
	Room	30.28% (*n* = 6,105)
	Other	6.8% (*n* = 1,372)
Socioeconomic stratum of the household***	1	1.42% (*n* = 287)
	2	0.83% (*n* = 168)
	3	30.28% (*n* = 6,105)
	4	6.8% (*n* = 1,372)
	5	1.42% (*n* = 287)
	6	0.83% (*n* = 168)
How scared are you of the following situations?		
Becoming sick or someone in your family becoming sick	Not scared	10.42% (*n* = 1,523)
		6% (*n* = 878)
		14.14% (*n* = 2067)
		13.64% (*n* = 1995)
	Very scared	55.8% (*n* = 8,160)
Not receiving medical attention if becoming sick from COVID-19 or something else	Not scared	7.48% (*n* = 1,093)
		5.04% (*n* = 737)
		12.43% (*n* = 1816)
		13.74% (*n* = 2008)
	Very scared	61.31% (*n* = 8,961)
Not working and/or not being able to pay bills	Not scared	11.32% (*n* = 1,635)
		4.66% (*n* = 673)
		10.88% (*n* = 1,572)
		11.79% (*n* = 1703)
	Very scared	61.35% (*n* = 8,863)
How often do you experience the following emotions and/or feelings?		
Boredom from being alone	Always	9.28% (*n* = 79)
	Almost always	10.93% (*n* = 93)
	Sometimes	28.08% (*n* = 239)
	Almost never	13.16% (*n* = 112)
	Never	38.54% (*n* = 328)
Anxiety from being alone	Always	7.82% (*n* = 66)
	Almost always	6.99% (*n* = 59)
	Sometimes	27.61% (*n* = 233)
	Almost never	13.27% (*n* = 112)
	Never	44.31% (*n* = 374)

How did you work during the pandemic?	
Not able to work at all	46.88% (*n* = 5,560)
Worked in-person	35.21% (*n* = 4,176)
Worked in-person then could not work	1.21% (*n* = 143)
Worked remotely	14.19% (*n* = 1,683)
Could not work and worked remotely	0.27% (*n* = 32)
Worked in-person and remotely	2.19% (*n* = 260)
Worked in-person; could not work and worked remotely	0.05% (*n* = 6)

*Sex: male 1, female 2.

**Colombia’s Sistema General de Seguridad Social en Salud (General System of Social Security in Health) proposes two modalities of affiliations to ensure health coverage for individuals and their families: the contributory regime for those who have the ability to pay and the subsidized regime for those who are in a condition of poverty or vulnerability.

***Socioeconomic stratification is the classification of housing, which is conducted in response to the regime of residential public services in Colombia (Law 142 of 1994). Strata 1, 2, and 3 correspond to people with fewer resources or who are beneficiaries of subsidies for residential public services; strata 5 and 6 correspond to people in upper strata with greater economic resources who must pay cost overruns (contribution) on the value of residential public services. Stratum 4 people are not beneficiaries of subsidies or required to pay cost overruns; the value that the company defines as the cost of providing the service is paid.

All participants who completed the survey were included. The exact number of individuals in the analysis was 20,535. Among eligible individuals from participating households, the median response rate was 90%, but ranged between 83 and 95% (9). While no participant was excluded from the analyses, pregnant women and people with specific pre-existing conditions were not included in the study. The total n in each of them varies depending on the lack of response to different questions.

### Data collection instrument

Based on the items of the general questionnaire (8), the main variables were defined, including feelings of fear, positive emotions, or feelings about the use of leisure time and work involvement, social vulnerability in the sociodemographic items (sex, age, ethnicity, socioeconomic stratum, place of residence), and job insecurity (unemployment, lack of social security). Five variables were constructed to determine the predictive profiles. The construction of each variable can be found in the supplementary information (Appendix 1 in S1 Supplementary materials), but their definitions are as follows:

#### Feelings of fear

This variable assesses a person’s level of fear of becoming ill, of not receiving medical care if ill, or of not working and/or not being able to pay financial obligations. The questions were answered on a scale of 1–5, where 1 was “not at all afraid” and 5 was “very afraid.” Internal consistency was excellent (omega = 0.94). A high score indicates that z person tends to be “very afraid” that he/she or someone in his/her family will become sick (p1), that he/she will not receive medical care if he/she becomes sick from COVID-19 or other causes (p2), and that he/she will not work and/or will not be able to pay his/her financial obligations (p3).

#### Positive emotions or feelings regarding the use of free time

This variable evaluates the frequency with which people experienced (p1) peace of mind, (p2) boredom due to a lack of occupation, and (p3) anxiety due to inactivity because of social isolation as a result of time off. The questions were answered on a scale from 1 to 5, where 1 was “always” and 5 was “never.” Internal consistency was excellent (omega = 0.87).

#### Positive emotions or feelings about living together

The three questions of this variable were answered only by the participants who lived with other people and were evaluated on a scale of 1 to 5, where 1 was “always” and 5 was “never.” This variable assessed the frequency with which people experienced “joy from having time to share with cohabitants” (p1), tiredness due to living with cohabitants (p2), and distress due to violent reactions from cohabitants (p3) as a result of living together during the period of social isolation. The internal consistency was excellent (omega = 0.85).

#### Positive emotions or feelings about loneliness

Composed of three questions answered only by those living alone and assessing the frequency of experiencing joy at being alone and having time for oneself (p1) and boredom (p2) and distress (p3) from being alone during the period of social isolation, this variable was rated on a scale of 1 to 5, where 1 was “always” and 5 was “never.” The internal consistency was excellent (omega = 0.90).

#### Work affectation during the period of social isolation

This variable consisted of two questions. The first item was “How much did you consider that your work was affected by the COVID-19 confinement measures?,” which was answered from 1 to 5, where 1 was “not affected at all” and 5 was “very affected.” The second item was “What has been your mode of work during the pandemic?.” This question was answered by participants who were not unemployed or who were students and/or pensioners who were not working. This variable originally consisted of three multiple response options: “Telework or virtual work” (Option 1), “In-person work” (Option 2), and “I could not work” (Option 3).

### Statistical analysis

Following the indications of Hair et al. (19), after verifying the fulfillment of the assumption of homoscedasticity in the analyzed profiles, to determine the classification statistic to be used (discriminant analysis or logistic regression), a binary logistic regression analysis calculated by the forward conditional stepwise method (20), because it was used to: (1) identify the predictive profile of each of the outcomes considered: emotional factors and the perceived impact on work due to confinement measures due to COVID-19, (2) addresses the problem of model overfitting in the sample data, because it only uses those variables that significantly improve the performance of the model are added. The polar extremes approach was used as a strategy to maximize primary variance (19), considering that the distribution of participants in all the outcomes studied did not follow an increasing monotonous (or isotonic) pattern; this strategy “helps because it is possible that group differences may appear even though the regression results are poor; that is, [...] can reveal differences that are not clear in a regression analysis of a complete set of data” (19). Qualitative predictor variables were also transformed into dummy variables (19). The qualitative adjustment of the model was determined based on the criteria of Hernández et al. (21, 22) for interpretation of the correlation (R) with respect to psychological tests. The effect size was analysed using Cohen’s 𝘧^2^ and its power (1-β) (23). We also analysed the size of the effect (ω) and the power (1-β) of the percentage of correct prediction, both global and specific, for each of the levels of the groups that compensate for the outcomes considered. Additionally, for - interpretation of the profiles, estimated coefficients (𝛽), which are called odds ratios (19), were analysed using SPSS Regression Models™ 16.0. (20). The models were built only with respondents to all variables.

### Ethical considerations

Individuals in selected households were invited to participate in the study. Upon acceptance, participants were presented with a consent form, which was read aloud to them, and signed by the participant and two witnesses. The consent form and research protocol were approved by the ethics committee of the Instituto Nacional de Salud (CEMIN 010/2020). We obtained written informed consent from each adult participant, as well as oral assent and written parental permission from participants aged 5–17.

## Results

### Predictive profile of life-threatening fear during the period of social isolation (quarantine) during the COVID-19 pandemic

The profile of participants (8,247 after excluding non-responses (NRs) in all variables considered for constructing the model, and 8,426 after excluding NRs only for predictor variables) responsible for answering the questions and successfully doing so can be deemed satisfactory, notwithstanding a few limitations (*R* = 0.32, *p* = 0.00). The overall correct classification rate stands at 62.4%. Within the “Low” group, the correct classification rate was 59.4%, whereas in the “High” group, it reached 65.4%. This indicates an intermediate-sized difference from chance in all instances (𝜔^overall^ = 0.25; 𝜔^Low^ = 0.31 and 𝜔^High^ = 0.19), and these findings hold statistical significance (1- 𝛽^overall^ = 1.00, 1- 𝛽^Low^ = 1.00 and 1- 𝛽^High^ = 1.00). It is worth noting that these results are applicable to both methods of assessing the fear of life-threatening situations during the period of social isolation (quarantine) amidst the COVID-19 pandemic (see Table 2).

**Table 2:** Regression coefficients and significance of the predictor variables of: life-threatening fear.

Variable	B	Sig.
Impact on work	0.21	0.000
Bogotá (0 = No; 1 = Yes)	−0.43	0.000
Cali (0 = No; 1 = Yes)	0.19	0.013
Cúcuta (0 = No; 1 = Yes)	0.67	0.000
Leticia (0 = No; 1 = Yes)	−0.19	0.043
Medellín (0 = No; 1 = Yes)	0.57	0.000
Villavicencio (0 = No; 1 = Yes)	0.50	0.000
Belonging to stratum 4 (0 = No; 1 = Yes)	−0.48	0.000
Belonging to stratum 5 (0 = No; 1 = Yes)	−0.73	0.000
Number of family members	0.23	0.003
Sex (1 = male and 2 = female)	0.38	0.000
White (0 = No; 1 = Yes)	0.37	0.000
Black (0 = No; 1 = Yes)	0.23	0.013
Employed (0 = No; 1 = Yes)	0.16	0.001
Number of cohabitants	−0.03	0.011
In-person and remote work (0 = No; 1 = Yes)	−0.44	0.004
Intercept	−1.56	0.000

Obtained from binary logistic regression analysis calculated by the forward conditional stepwise method. Ependent variable: life-threatening fear during the period of social isolation (quarantine) during the COVID-19 pandemic (0 = low/1 = high).

The following profile suggests that a person with such characteristics had a greater probability of experiencing a high level of life-threatening fear of COVID-19 during the period of social isolation (quarantine). Women (𝛽 = 0.39; *p* = 0.00); also, participants who self-identification as Black (𝛽 = 0.27; *p* = 0.00) or White (𝛽 = 0.39; p = 0.00); shown an increased risk. Also, residents in ere (𝛽 = −0.49; *p* = 0.00) or stratum 5 (𝛽 = −0.51; *p* = 0.00); participants living only with family members (𝛽 = 0.27; *p* = 0.00); Fewer people living in the dwelling (𝛽 = −0.04; *p* = 0.00)During the last 12 months, before participating in the study, the main occupation was working as an employee (𝛽 = 0.16; *p* = 0.00); At the time of the research, the participant was not insured by any health regime (𝛽 = 0.21; *p* = 0.05); Worked in person and remotely (𝛽 = −0.50; *p* = 0.00) during confinement; had a greater perception that his/her work was impacted by COVID-19 confinement measures (𝛽 = 0.21; *p* = 0.00); and Not living in the city of Bogotá (𝛽 = −0.43; *p* = 0.00) or Leticia (𝛽 = −0.19) or living in Cali (𝛽 = 0.20; *p* = 0.01), Cúcuta (𝛽 = 0.68; *p* = 0.00), Medellín (𝛽 = 0.57; *p* = 0.00), or Villavicencio (𝛽 = 0.51; *p* = 0.00).

Otherwise, individuals had a greater probability of having experienced lower life-threatening fear during the period of social isolation due to COVID-19.

### Predictive profile of positive emotions or feelings regarding the use of free time

The profile is composed of 16 variables, 11 of which were dichotomized based on (i) city of residence, (ii) socioeconomic stratum of the household, (iii) Household composition, (iv) self-perceived race based on culture, people, or physical traits, (v) health regime, and (vi) modality of work during the pandemic (14). The sample for this profile is composed of 8,232 participants (after eliminating the NRs for all the variables considered for construction of the model) and 8,300 (after eliminating the NRs only for the predictor variables); that is, the number of participants was reduced by 47.72% (*n* = 6,853).

The profile obtained can be considered adequate despite some shortcomings (*R* = 0.25; *p* = 0.00), with an overall percentage of correct classification of 59.1%. For the “Low” group, the percentage of correct classification was 52.0%, and for the “High” group, the percentage of correct classification was 65.5%, implying a difference of intermediate size with respect to chance in the overall prediction and in the prediction for the “High” group (𝜔_overall_ = 0.18; 𝜔_High_ = 0.31). In the prediction for the “Low” group, the size difference was small (𝜔_Low_ = 0.04) with respect to chance. In all cases, the prediction was statistically powerful (1-𝛽_overall_ = 1.00, 1-𝛽_Low_ = 1.00 and 1-𝛽_High_ = 1.00). Therefore, the interpretation applies only to the “High” group for positive emotions or feelings during free time because the prediction exceeds the limit of chance.

In this sense and considering that the group classified as “Low” (N_Low_ = 3,590) was coded as 0 and the group classified as “High” (N_High_ = 3,918) was coded as 1, the following profile indicates that a person with such characteristics had a greater probability of experiencing a high level of positive emotions or feelings during free time (See Table 3).

**Table 3:** Regression coefficients and significance of the predictor variables of: free time use.

Variable	B	Sig.
Barranquilla (0 = No; 1 = Yes)	−0.194	0.027
Bogotá (0 = No; 1 = Yes)	−0.448	0.000
Bucaramanga (0 = No; 1 = Yes)	0.441	0.000
Cúcuta (0 = No; 1 = Yes)	0.311	0.001
Ipiales (0 = No; 1 = Yes)	0.281	0.001
Leticia (0 = No; 1 = Yes)	−0.366	0.000
Belonging to stratum 1 (0 = No; 1 = Yes)	−0.187	0.002
Number of family members	0.210	0.006
Age	0.006	0.000
Other race (0 = No; 1 = Yes)	−0.288	0.001
Unknown race (0 = No; 1 = Yes)	−0.455	0.000
Employed (0 = No; 1 = Yes)	0.127	0.011
Healthcare system: Contribution (0 = No; 1 = Yes)	0.181	0.000
Number of cohabitants	−0.021	0.035
In-person or remote work (0 = No; 1 = Yes)	0.415	0.007
Perceived impact on work due to COVID-19 confinement	−0.157	0.000
Intercept	0.242	0.054

Obtained from binary logistic regression analysis calculated by the forward conditional stepwise method. Ependent variable: positive emotions or feelings regarding the use of free time (0 = low/1 = high).

We found (𝛽 = 0.01; *p* = 0.00); self-recognition as belonging to an ethnic group (𝛽 = −0.33; *p* = 0.00), including the Mestizo, White, Black, or indigenous race (𝛽 = −0.46; *p* = 0.00); Dwelling not in an area classified as stratum 1 (𝛽 = −0.19; *p* = 0.00); A household composed only of family members (𝛽 = 0.22; *p* = 0.01), with a greater number of families living in the dwelling (𝛽 = 0.12; *p* = 0.01) but a smaller number of people within the household (𝛽 = −0.03; *p* = 0.01); Worked as an employee during the last 12 months before participating in the study (*B* = 0.13); An opportunity to work in person and remotely during the pandemic (𝛽 = 0.39; *p* = 0.01); Insured by a contributory health regime at the time of conducting the research (𝛽 = 0.22; *p* = 0.00); No strong perception that work was impacted by confinement measures (𝛽 = −0.15; *p* = 0.00); Not living in the city of Barranquilla (= −0.16; *p* = 0.08), Leticia (𝛽 = −0.37), or Bogotá (𝛽 = −0.46; *p* = 0.00) or living in Bucaramanga (𝛽 = 0.46; *p* = 0.00), Cúcuta (𝛽 = 0.33; *p* = 0.00), or Ipiales (𝛽 = 0.30; *p* = 0.00); and Better informed about coronavirus symptoms as demonstrated by identifying a greater number of symptoms (𝛽 = 0.03; *p* = 0.02).

However, with a profile opposite to the previous one, there was no margin of probability beyond chance that the person will experience a low level of positive emotions or feelings during their free time.

### Predictive profile of the perceived impact on work due to COVID-19 confinement measures

The profile is composed of 10 variables, eight of which were dichotomized (dummy variables) based on (i) the socioeconomic stratum of the household, (ii) main occupation during the last 12 months, and (iii) Modality of work during the pandemic. The sample for this profile is composed of 4,284 participants (after eliminating the NRs for all the variables considered for construction of the model) and 4,573 (after eliminating the NRs only for the predictor variables) who were responsible for answering such questions and successfully did so.

The profile obtained can be considered good (*R* = 0.47; *p* = 0.63), with an overall percentage of correct classification of 70.4% For the “Not affected” group, the percentage of correct classification was 48.7%, and for the “Very affected” group, the percentage of correct classification was 81.5%, which implied the following: in the overall prediction, a difference of intermediate size that was statistically powerful with respect to chance (𝜔_overall_ = 0.41; 1-𝛽_overall_ = 1.00); in the “Very affected” group, a large difference with respect to chance (𝜔_High_ = 0.63; 1-𝛽_High_ = 1.00); and in the “Not affected” group, a small difference (𝜔_Low_ = 0.03) that was not powerful (1-𝛽_Low_ = 0.07) with respect to chance.

The above findings assume that the interpretation applies only to the group that was “Very affected” in their work by confinement measures because the prediction exceeds the limit of chance. In this sense and considering that the group classified as “Not affected” (N_Low_ = 224) was coded as 0 and the group classified as “Very affected” (N_High_ = 439) was coded as 1, the following profile indicates that a person with such characteristics was more likely to experience their work being greatly affected by COVID-19 confinement measures (See Table 4).

**Table 4:** Regression coefficients and significance of the predictor variables of: work impact.

Variable	B	Sig.
Cali (0 = No; 1 = Yes)	−0.62	0.000
Cúcuta (0 = No; 1 = Yes)	−0.34	0.016
Medellín (0 = No; 1 = Yes)	−0.55	0.000
Age	−0.01	0.003
Race: Indigenous (0 = No; 1 = Yes)	0.40	0.031
Marital status: Partnered (0 = No; 1 = Yes)	0.28	0.000
Employed (0 = No; 1 = Yes)	0.81	0.000
Employment status: Independent (0 = No; 1 = Yes)	1.63	0.000
Employment status: Informal (0 = No; 1 = Yes)	1.39	0.000
Employment status: Pensioned (0 = No; 1 = Yes)	−0.59	0.010
Healthcare System: Contribution (0 = No; 1 = Yes)	−0.59	0.000
Number of symptoms	0.22	0.000
Not able to work at all (0 = No; 1 = Yes)	1.26	0.000
In-person work (0 = No; 1 = Yes)	1.41	0.000
Remote work (0 = No; 1 = Yes)	2.47	0.018
Financial assistance (0 = No; 1 = Yes)	0.25	0.007
Intercept	−0.92	0.000

Obtained from binary logistic regression analysis calculated by the forward conditional stepwise method. Ependent variable: perceived impact on work due to COVID-19 confinement measures (0 = Not affected/1 = Very affected).

For people with younger age (𝛽 = −0.01); Marital status of Common-Law Marriage (𝛽 = 0.28); Indigenous (𝛽 = 0.40); Main occupation as an independent worker (𝛽 = 1.06; *p* = 0.02), working without a pension (𝛽 = −1.75; *p* = 0.01), working as an employee (𝛽 = 0.81), or working as an informal worker (𝛽 = 1.39) during the last 12 months; Inability to work at any time (𝛽 = 1.45; *p* = 0.00), worked in person for a while but then could not work (𝛽 = 1.91; *p* = 0.01), or were not working for a while and then worked remotely (𝛽 = 21.31; *p* = 1.00) during the pandemic; Received financial assistance during the period of confinement (𝛽 = 0.25); Not insured under the contributory health regime at the time of conducting the research (𝛽 = −0.59); Not living in the cities of Cali (𝛽 = −0.62), Cúcuta (𝛽 = −0.34), or Medellín (𝛽 = −0.55); and Better informed about coronavirus symptoms as demonstrated by identifying a greater number of symptoms (𝛽 = 0.12; *p* = 0.03).

However, with a profile opposite to the previous profile, there was no margin of probability beyond chance that the person will experience a low level of positive emotions or feelings during free time.

## Discussion

### Psychosocial impact and vulnerability

In a social impact report that precedes this article, the descriptive data had already revealed a detrimental effect of the COVID-19 lockdown period on the mental well-being of vulnerable population groups (9). The findings of this study imply a connection among all the variables being investigated, including the fear of life-threatening situations, leisure time utilization, and the impact on employment. These correlations appear to be influenced by social vulnerability, which is determined by sociodemographic factors such as gender, age, ethnicity, and socioeconomic status. Working conditions, particularly job insecurity, seem to play a significant role in this relationship, with those who are unemployed, lacking social security, and perceiving a high impact on their work experiencing greater vulnerability.

Furthermore, the unequal impact observed across different cities may be associated with the epidemiological trajectory of each city at the time of data collection (9) or structural vulnerability factors inherent to the study locations, such as access to healthcare services. These factors could have contributed to the divergent psychosocial impact during the pandemic (24). However, a discernible pattern defined by city type, region, or level of development did not emerge, leaving room for exploration in future studies.

For the variable life-threatening fear, people with high vulnerability factors presented a high level of fear. Working conditions were the main factor; a negative impact was found for employed people without social security who could not work during confinement and felt that their work was strongly impacted. Women and individuals in middle and low socioeconomic strata (less than or equal to 3) were more affected by sociodemographic factors. With respect to structural vulnerability, cities with the highest levels of fear (Cúcuta, Medellín, Villavicencio, and Cali) had very low, low, and intermediate levels of potential access (25) to health services at the regional level and, at the time of data collection, a medium level of infections. In contrast, Bogotá, where residents reported the lowest level of fear of life, had a very high level of access to health services, and at the time of data collection, the three cities with residents with the lowest levels of fear (Leticia, Barranquilla, and Bogotá) had low levels of infection.

With respect to the variable free time, the data suggest a relationship between a high level of positive emotions or feelings during free time and low vulnerability factors, which is mainly evident in employment vulnerability factors, positively affecting people who were able to work remotely or in person, perceived a low impact on work due to the pandemic, and had social security during confinement. A positive impact for sociodemographic factors was found for adults who recognized themselves within an ethnic group, including Mestizo, White, Black, or indigenous, and did not belong to the most vulnerable stratum (3). In contrast, neither negatively affected cities (Bogotá, Barranquilla, and Leticia) nor positively affected cities (Bucaramanga, Cúcuta, and Ipiales) presented a defined pattern related to the epidemiological curve, and no other related structural factors were found.

With respect to the variable work impact, the data suggest a relationship between perceiving a high work impact and high vulnerability factors, which is mainly reflected in terms of employment vulnerability because it negatively affected those who could not work during the entire period of confinement or only part of it, those who were informal workers, and those receiving financial assistance during confinement or were not pensioners and were not part of the contributory health regime; however, employed workers were also affected. A lower impact of sociodemographic factors was found for people who recognized themselves as indigenous. With respect to the cities, Cali, Cúcuta, and Medellín had residents who reported the least impact on work, possibly because at the time of data collection, they had medium levels of infection.

### Risk factors and care measures for future health emergencies

The predictive capacity of the variables studied allows the identification of key risk factors for coping with future emergencies and implementing different measures that mitigate the negative psychosocial impacts and in turn promote behaviours that favour positive reactions to a crisis situation.

The data suggest that the main risk factor was working conditions, a factor that was positively or negatively related to the three study variables; that is, people who could not work partially or completely during confinement experienced negative psychosocial impacts, while in contrast, those who could work remotely or in person showed positive psychosocial factors. In terms of the sociodemographic factors, race was found to be a risk factor that affected the three variables (although a particular ethnic group was not clearly defined), as was socioeconomic stratum, which negatively affected two of the three study variables, indicating that people who belonged to low and middle socioeconomic strata (1, 2 and 3) had a greater risk of negative psychosocial effects. Last, place of residence was identified as a risk factor for two of the three variables, indicating that the risk of negative psychosocial effects is different for each city.

According to the above data, the following general recommendations or guidelines are proposed for future emergencies:

Although containment measures in the face of a health emergency usually have economic implications, measures with the least possible social-employment impacts should be sought, and severe containment measures such as confinement should be avoided. As has occurred in many middle- or low-income countries such as Colombia, the high degree of informal employment, which was 55% in 2020, the absence of unemployment benefits, and the limited scope of emergency financial assistance (85% of people did not receive financial assistance) increased the economic impact of the confinement measures, which had brought negative psychosocial implications (8). High-impact socioeconomic problems are associated with a high level of risk of negative psychosocial impacts and an increase in social problems, which can manifest as violence. The results from several studies in the Colombian context suggest that socioeconomic and psychosocial effects are related to social problems of great magnitude, such as the national strike that occurred in May 2021 (26–28).

To mitigate these socioeconomic impacts, different studies (29, 30) propose a scale of health containment measures that would be implemented based on the magnitude of the emergency: in the initial phases, mass activities are restricted, and protection measures such as face masks are promoted; in intermediate phases, work activities are limited, and remote work and flexible work schedules are emphasized; and extreme measures of total confinement should be implemented only in maximum health alert phases, with financial subsidies for the most vulnerable groups. Likewise, the differential behaviour of psychosocial impacts in each city shows the need for the implementation of differential actions at the regional level (29, 30) that consider the degree of the local emergency, structural problems, and socio-institutional resources.

The psychosocial impacts as a result of a crisis situation requires prevention and recovery actions in terms of ensuring timely psychosocial and mental health care, both in the initial phases of negative impacts and in advanced high-risk situations to avoid potential criminal or suicidal behaviour. Access to these psychosocial care measures must be guaranteed, creating necessary mechanisms such as remote or telephone assistance in the event that emergency measures do not allow face-to-face care and must be free of charge to ensure elimination of economic barriers to access (31). Likewise, such psychosocial care should be focused mainly on people with identified risk factors, such as unemployed individuals, the poorest social groups, and racial minorities, and as the report preceding this article and other studies suggest, older adults and the young population should be prioritized (10).

Finally, as prevention measures, some studies note the importance of the design and implementation of work, educational, and recreational adaptation programmes that prevent the negative psychosocial impacts caused by poorly managed fear, anxiety, stress, frustration, depression, and various situations of conflict derived from an emergency and its containment measures (32–34). Therefore, the following measures must be developed: (i) guidelines on behaviour, coexistence, and protection for people who must continue working because of their importance in the entire chain of essential supplies and for health personnel designed for these workers, their employers, and society in general; (ii) work adaptation programmes and virtual and distance-learning modalities for the entire population; and iii. Specialized programmes with special educational and recreational activities for various population segments differentiated by age, sex, and race, among others.

All these mitigation and prevention measures require three key factors for their operation: (i) Ensuring internet access for all populations, as this could become a factor of exclusion from the adaptation and protection measures proposed; (ii) activation of community and local networks for those who are excluded from the virtual world due to cultural, schooling, or age limitations; and (iii) Studying and evaluating comprehensive strategies to identify vulnerability and deliver services or subsidies, since these will be indispensable in cases of total loss of income sources for the most vulnerable groups on a long-term basis.

## Limitations

Our research possesses several notable strengths, such as the use of probabilistic participant sampling, high participation rates, and the utilization of a serological test with a proven track record of exceptional sensitivity and specificity in validation studies. However, it is essential to acknowledge the study’s limitations as well. Firstly, although we conducted the study in several densely populated areas and border cities of Colombia, the findings cannot be extrapolated to unsampled populations or the entire country. Notably, our research excluded rural and institutionalized populations, which may have experienced distinct epidemic patterns different from those observed here. Additionally, pregnant women and individuals with specific pre-existing conditions were not included in the study.

Secondly, because the study spanned a three-month period, differences observed between cities could be attributed to timing. Some cities enrolled in the study were already entering a second epidemic peak, possibly resulting in higher seroprevalence compared to cities sampled earlier. To accurately gauge the current seroprevalence levels in these populations, especially since most included cities experienced epidemic peaks after our study, additional serosurvey rounds would be necessary.

## Conclusion

Negative psychosocial impacts during the period of confinement have a strong relationship with social vulnerability given working conditions and sociodemographic characteristics and possibly with the level of emergency differentiated by city. The main risk factor for negative psychosocial impacts was the loss of work during the entire period of confinement or part of it, followed by belonging to vulnerable ethnic groups, socioeconomic strata and the place of residence.

Based on the aforementioned findings, the primary guidelines for addressing future health emergencies are as follows: (i) Mitigation of socio-employment impacts through emergency containment measures according to the contagion curve on a local level, implementing total confinement measures only in phases of maximum health alert, including economic subsidies for the most vulnerable groups; (ii) Prevention and recovery efforts through accessible psychosocial and mental health care for the entire population, with particular focus on vulnerable groups; (iii) Development and implementation of work, educational, and recreational programs that facilitate the adaptation of people to the changes brought by confinement processes.

## Data availability statement

The raw data supporting the conclusions of this article will be made available by the authors, without undue reservation.

## Ethics statement

The studies involving humans were approved by Ethics and Research Committee [(CEMIN 010/2020)] of the Instituto Nacional de Salud (Colombia). The studies were conducted in accordance with the local legislation and institutional requirements. The participants provided their written informed consent to participate in this study.

## Author contributions

MB-G: Conceptualization, Formal analysis, Writing – original draft. AM: Methodology, Writing – original draft. JP: Conceptualization, Formal analysis, Writing – original draft. LZ: Resources, Writing – original draft. DB: Resources, Writing – original draft. YT: Methodology, Writing – review & editing. JM-R: Methodology, Writing – review & editing. MM-R: Resources, Writing – review & editing. MO: Resources, Writing – review & editing.
