# Supplementary material for: Psychosocial risk profiles to address future health emergencies: a country study during the COVID-19 lockdown period in Colombia
Source: Front Public Health. 2024 Mar 22;12:1323490. doi: 10.3389/fpubh.2024.1323490 (PMC11008715; doi:10.3389/fpubh.2024.1323490)
Supplement: Supplementary file 1 [file Data_Sheet_1.docx]

**S1. Supplementary materials**

**Appendix 1. Tools.**

**Factors related to vital fear during the period of social isolation (quarantine) due to COVID-19:** Composed of three questions evaluating the level of fear of becoming sick, not receiving medical attention in case of illness, and not working and/or not being able to fulfil financial obligations. The questions could be answered on a scale of 1 to 5, where 1 was “no fear” and 5 was “a lot of fear”. Likewise, the internal consistency was excellent (𝛺=0.94). In this sense, a person with a maximum score for this factor tends to have “a lot of fear” of becoming sick or someone in his/her family becoming sick (p_1_), fear of not receiving medical attention in case of becoming sick with COVID-19 or other illnesses (p_2_), and fear of not being able to work and/or fulfil financial obligations” (p_3_).

Such scores (n=14,376) were interpreted at two prevalence levels, “Low” (P_Bajo_=50.62%) and “High” (P_Alto_=50.62%), when choosing the extreme values of the variable (Arnau, 1979, p. 153) as a strategy to maximize the primary variance due to the following: 1) the factorial distribution strongly leaned towards its lower end (As=-1.31; 𝛿_As_=0.02) and was dramatically flattened (Ku=0.56; 𝛿_Ku_=0.04), which hinder the presence of a normal distribution (p_K-S_=0.00), as well as any other distribution whose form facilitates the creation of a predictive profile; and 2) no subject had a score that coincided with the exact value of the median, which was the parameter used to achieve an objective, mutually exclusive, and balanced classification (𝜔=0.01; p=0.14 and 1-𝛽=0.13).

**Factors related to emotions or positive feelings regarding the use of free time during the period of social isolation (quarantine) due to COVID-19:** Composed of three questions evaluating the frequency of tranquillity during rest time (p1), boredom due to a lack of occupation (p2), and anxiety due to inactivity (p3) considering time use during the social isolation period caused by the health contingency of COVID-19, which were rated on a scale of 1 to 5, where 1 was “always” and 5 was “never”. Likewise, the internal consistency was excellent (𝛺=0.87). In this sense, a person will have a maximum score if he/she answers that he/she “always” (1) had “tranquillity during rest time” (p1), “never” (5) felt “boredom due to a lack of occupation in his/her free time” (p2), and “never” (5) felt “anxiety due to inactivity” (p3).

Such scores (n=14,360) were interpreted at two prevalence levels, “Low” (P_Bajo_=49.30%) and “High” (P_Alto_=50.70%), when choosing the extreme values of the variable (Arnau, 1979, p. 153) as a strategy to maximize the primary variance due to the following: 1) the factorial distribution strongly leaned towards its lower end (As=-0.41; 𝛿As=0.02) and was dramatically flattened (Ku=-0.93; 𝛿Ku=0.04), which hinder the presence of a normal distribution (pK-S=0.00), as well as any other distribution whose form facilitates the creation of a predictive profile; and 2) no subject had a score that coincided with the exact value of the median, which was the parameter used to achieve an objective, mutually exclusive, and balanced classification (𝜔=0.01; p=0.10 and 1-𝛽=0.39).

**Factors related to positive emotions or feelings regarding living together during the period of social isolation (quarantine) due to COVID-19:** Composed of three questions answered only by people who lived with several people at their home, this tool evaluated the frequency with which they experienced joy in relation to having time to share with cohabitants (p1), tiredness due to cohabitation with the people with whom they live (p2), and anguish due to violent reactions of the people with whom they live (p3) as a result of cohabitation during the period of social isolation due to the health contingency of COVID-19, which were rated on a scale of 1 to 5, where 1 was “always” and 5 was “never”. Likewise, the internal consistency was excellent (𝛺=0.85). In this sense, a person would obtain the maximum score if they answered that he/she “always” (1) had “joy in relation to having time to share with cohabitants” (p1), that he/she “never” (5) felt “tired due to cohabitating with people with whom they live” (p2), and that he/she “never” (5) felt “anguish due to violent reactions of the people with whom they live” (p3).

Such scores (n=13,545) are interpreted at two prevalence levels, “Low” (P_Bajo_=51.44%) and “High” (P_Alto_=48.56%), when choosing the extreme values of the variable (Arnau, 1979, p. 153) as a strategy to maximize the primary variance due to the following: 1) the factorial distribution strongly leaned towards its lower end (As=-1.05; 𝛿As=0.02) and was dramatically flattened (Ku=-0.22; 𝛿Ku=0.04), which hinder the presence of a normal distribution (pK-S=0.00), as well as any other distribution whose form facilitates the creation of a predictive profile; and 2) no subject had a score that coincided with the exact value of the median, which was the parameter used to achieve an objective, mutually exclusive, and balanced classification (𝜔=0.03), although the difference was significant (p=0.00;1-𝛽=0.92).

**Factors related to emotions or positive feelings regarding loneliness during the period of social isolation (quarantine) due to COVID-19:** Composed of three questions answered only by people who lived alone, this tool evaluated the frequency with which they experienced joy regarding being alone and having time for himself (p1) and boredom (p2) and anguish (p3) in relation to being alone during the period of social isolation due to the sanitary contingency of COVID-19, which were rated on a scale of 1 to 5, where 1 was “always” and 5 was “never”. Additionally, the internal consistency was excellent (𝛺=0.90). In this sense, a person would have a maximum score if he/she answered that he/she “always” (1) had “joy towards being alone and having time for himself” (p1), that he/she “never” (5) experienced “boredom when alone” (p2), and that he/she “never” (5) experienced “anxiety because of loneliness” (p3).

Such scores (n=843) are interpreted at two prevalence levels: “Low” (P_Bajo_=48.40%) and “High” (P_Alto_=51.60%), when choosing the extreme values of the variable (Arnau, 1979, p. 153) as a strategy to maximize the primary variance due to the following: 1) the factorial distribution strongly leaned towards its lower end (As=-0.50; 𝛿As=0.08) and was dramatically flattened (Ku=-0.62; 𝛿Ku=0.17), which hinder the presence of a normal distribution (pK-S=0.00), as well as any other distribution whose form facilitates the creation of a predictive profile; and 2) no subject had a score that coincided with the exact value of the median, which was the parameter used to achieve an objective, mutually exclusive, and balanced classification (𝜔=0.03; p=0.35; 1-𝛽=0.15).

**Job-related affectations during the period of social isolation (quarantine) due to COVID-19:** A single question rated from 1 to 5, where 1 was “not affected” and 5 was “very affected”, evaluating how much a person considered that his/her work was affected by the COVID-19 confinement measures. According to the CIDEIM/UN technical report, the participants showed a characteristic prevalence of “very affected” (p=34.17%, 1-𝛽=1.00, ω=0.40, p𝜒²=0.00).

**Which has been your working modality during the pandemic?** This question was answered by 11,859 participants who were not unemployed or who were students and/or pensioners who were not working. Originally composed of three multiple response options: “Telework or virtual work” (Option 1), “Face-to-face work” (Option 2), and “I could not work” (Option 3), the combination of which generated seven different working modalities; the statistical majority reported that “I couldn’t work at any time” (p=46.88%, 1-𝛽=1.00, ω=0.78, p𝜒²=0.00).

**Procedure**

The information was collected between November 2020 and December 2020 (INS, 2021). Data collection was implemented face to face at the participants’ homes. The invitation to participate was made by home visit or by telephone in random order, and when the households visited did not respond, they were visited three more times before inviting neighbouring households to participate (Mercado, et al, 2020). RedCap‍ V.10.1.2 (free licence) was used to record geographic information and participants’ responses (Mercado, et al, 2020, p.3).

**Appendix 2. Participants.**

The sample was composed of 20,535 people and 7,436 households surveyed in the 10 cities of the study; however, because not all the questions in the questionnaire were answered, the sample size varied with respect to the questions referring to emotions during preventive isolation due to COVID-19 and its effect on work as follows: 13,360 people for the analysis of emotions during free/leisure time and 14,376 people for the analysis of fear of situations due to COVID-19, emotions about coexistence, and loneliness during confinement due to COVID-19. The origin of both samples was 7,159 households. With respect to the level of job-related affectation, the sample was composed of 15,010 people from 7,222 households (for greater precision on the descriptive characteristics of the sample).
